# Ozone uptake at night is more damaging to plants than equivalent day-time flux

**DOI:** 10.1007/s00425-021-03580-w

**Published:** 2021-02-24

**Authors:** Eleni Goumenaki, Ignacio González-Fernández, Jeremy D. Barnes

**Affiliations:** 1 Plant and Microbial Biology, School of Natural and Environmental Science [SNES], Devonshire Building, Newcastle University, Newcastle Upon Tyne, NE1 7RU UK; 2 School of Agricultural Sciences, Hellenic Mediterranean University, P.O. Box 1939, GR-71004 Heraklion, Crete Greece; 3 Ecotoxicology of Air Pollution, CIEMAT, Avda. Complutense, 40.28040 Madrid, Spain

**Keywords:** Diel ozone sensitivity, Night-time exposure, Ozone detoxification and repair, Air pollution, Climate change

## Abstract

**Main conclusion:**

Plants exposed to equivalent ozone fluxes administered during day-time versus night-time exhibited greater losses in biomass at night and this finding is attributed to night-time depletion of cell wall-localised ascorbate.

**Abstract:**

The present study employed *Lactuca sativa* and its closest wild relative, *L. serriola*, to explore the relative sensitivity of plants to ozone-induced oxidative stress during day-time versus night-time. By controlling atmospheric ozone concentration and measuring stomatal conductance, equivalent ozone uptake into leaves was engineered during day and night, and consequences on productivity and net CO_2_ assimilation rate were determined. Biomass losses attributable to ozone were significantly greater when an equivalent dose of ozone was taken-up by foliage at night compared to the day. Linkages between ozone impacts and ascorbic acid (AA) content, redox status and cellular compartmentation were probed in both species. Leaf AA pools were depleted by exposure of plants to darkness, and then AA levels in the apoplast and symplast were monitored on subsequent transfer of plants to the light. Apoplast AA appeared to be more affected by light–dark transition than the symplast pool. Moreover, equivalent ozone fluxes administered to leaves with contrasting AA levels resulted in contrasting effects on the light-saturated rate of CO_2_ assimilation (*A*_sat_) in both species. Once apoplast AA content recovered to pre-treatment levels, the same ozone flux resulted in no impacts on *A*_sat_. The results of the present investigation reveal that plants are significantly more sensitive to equivalent ozone fluxes taken-up at night compared with those during the day and were consistent with diel shifts in apoplast AA content and/or redox status. Furthermore, findings suggest that some thought should be given to weighing regional models of ozone impacts for extraordinary night-time ozone impacts.

## Introduction

There is growing concern over the impacts of rising tropospheric ozone levels on crop yield in many parts of the world, since this pollutant is likely to continue to negatively influence food supplies for the foreseeable future (Mills et al. 2018; Ainsworth et al. 2020). Exposure to elevated levels of ozone results in detrimental effects on vegetation following uptake into plant foliage via open stomata, where it rapidly decomposes in the cell walls of leaf mesophyll cells to yield a host of Reactive Oxygen Species (ROS; Agathokleous et al. 2020; Foyer et al. 2020). This sequence of events propagates a dramatic and resource-expensive shift in gene expression, reflected in a decline in photosynthetic capacity, retention of fixed carbon in source leaves and the up-regulation of repair and defence networks which is ultimately manifested in reduced productivity (Goumenaki et al. 2010; Gillespie et al. 2015).

Ozone pollution is generally perceived as a day-time problem, since the gas is generated as a result of complex photolytic reactions involving nitrogen oxides and volatile organic carbons and the stomata of most plants are commonly open during the day under favourable conditions, enabling ozone uptake into foliage (Royal Society 2008). However, there are many situations, where elevated ozone concentrations are also experienced at night concurrent with situations where plants may exhibit significant stomatal conductance, for example during significant episodes in the Mediterranean region (Kourtidis et al. 2002; Mavrakis et al. 2010; Kopanakis et al. 2016) and in the uplands and wetlands in other areas (Elvira et al. 2016; Warmiński and Bęś 2018). Moreover, night-time ground-level ozone concentrations are reported to be increasing in areas, where NO_x_ emissions are rising, as part of a global change in the diurnal cycle of the pollutant (Strode et al. 2019). It is widely reported that exposure to ozone pollution reduces stomatal perception and response to environmental factors. This is known as ‘stomatal sluggishness’, a condition that persists through the night in multiple species (Paoletti and Grulke 2010; Hoshika et al. 2016; Grulke and Heath 2020). This sluggishness causes additional ozone uptake and reduced water use efficiency (Emberson 2020) and maybe influenced by nutrient supply (Hoshika et al. 2019). Indeed, there is growing recognition that night-time stomatal ozone uptake maybe significant in many wild and cultivated species (Caird et al. 2007; Mereu et al. 2009; González-Fernández et al. 2010; Grantz et al. 2016).

Under occasional conditions, where ozone concentration remains elevated at night it is important to know whether ozone taken-up into the leaf interior at night is significantly more damaging than the equivalent flux taken up during the day. This is also a key factor requiring dial-in to models relating to ozone impacts on vegetation, particularly in the Mediterranean region (Millán et al. 2000; Goumenaki et al. 2007; Pleijel et al. 2007; Grünhage et al. 2012; González-Fernández et al. 2013, 2014; Elvira et al. 2016; Querol et al. 2018). Current flux-based ozone risk assessment models, adopted by the UNECE Convention on Long-Range Transboundary Air Pollution for negotiating air pollution abatement protocols, assume minimal night time stomatal conductance (CLRTAP 2017).

A principal factor controlling ozone tolerance is believed to be the composition of the cell wall matrix bounding leaf mesophyll cells, and in particular the level, regeneration rate and redox status of ascorbic acid (AA) in this sub-cellular compartment (Plöchl et al. 2000; Turcsànyi et al. 2000; Sanmartin et al. 2003; Yendrek et al. 2015). The level and redox status of AA in the leaf apoplast may combat ozone-induced oxidative stress through direct quenching of the resulting ROS and/or the regulation of defence-related gene expression (Noctor et al. 2018). The dynamics of AA catabolism in the apoplast, and transfer from the cytosol, probably play a critical role in determining plant responses to ozone stress (Barnes et al. 2002; Vainonen and Kangasjärvi 2015; Dai et al. 2020) and have been revealed by QTL pyramiding exercises to be amongst the most important and distinct genetic traits governing the tolerance of rice (Frei et al. 2008, 2010) and wheat (Feng et al. 2010; Barnes and Quarrie, unpublished) to elevated levels of ozone. Ascorbate levels in cellular and subcellular compartments are the product of net biosynthesis, recycling, degradation/oxidation and/or intercellular and intracellular transport (Mellidou and Kanellis 2017; Foyer et al. 2020). Light, as well as other environmental factors, are known to directly regulate ascorbic acid metabolic pathways (Ntagkas et al. 2018). Exposure of lettuce to contrasting light regimes results in the upregulation of transcript levels of *GMP* (GDP-d-mannose pyrophosphorylase), *GME* (GDP-d-mannose 3'5-epimerase), *GGP* (GDP-L-galactose phosphorylase), *GPP* (L-galactose-1-phosphate phosphatase), and *GLDH* (L-galactono-1,4-lactone dehydrogenase) which encode key enzymes in the GDP-L-galactose phosphorylase and GDP-D-mannose-3,5-epimerase biosynthetic pathways (Zha et al. 2020).

Cell wall-localised AA content has been shown to be correlated with O_3_ tolerance in several studies (Lyons et al. 1999; Turcsànyi et al. 2000; Maddison et al. 2002; Padu et al. 2005). This contention is also supported by research on the targeted transgenic manipulation of apoplast ascorbate content/redox status (Sanmartin et al. 2003; Pignocchi et al. 2006; Ueda et al. 2015). Current views suggest that it  probably is the regulation of AA content in the apoplast that modulates responses to ozone-induced oxidative stress rather than AA content per se (Zechmann 2018) and highlight the need for further research related to AA involvement in plant responses and recovery mechanisms after detrimental exposure (Bellini and De Tullio, 2019).

There is little information on diel variations in cell wall-localised AA content. However, Moldau and co-workers (1998) reported a marked decline in AA content at night-time in barley (*Hordeum vulgare*), and it is known that transfer of plants from high to low irradiance results in marked and rapid shifts in leaf AA content (Gest et al. 2013), expected to be mirrored in shifts in the cell wall-localised AA pool. This raises the question as to whether ozone uptake at night is especially damaging for plants.

In the present study, we probed the linkage between AA content, redox status of the leaf apoplast (and symplast) and ozone injury in *L. sativa* cv. Paris Island, as well as its closest wild relative, *L. serriola* to investigate any intrinsic interspecific variation. Light levels were used to manipulate sub-cellular pools of AA. Plants were exposed to prolonged darkness to reduce AA in the leaf cell walls. Shifts in AA were monitored on subsequent transfer of plants to the light. Stomatal conductance was measured in parallel with atmospheric ozone exposure, and this was used to deliver equivalent ozone fluxes to leaves with contrasting AA levels. Ozone impacts were assessed via impacts on growth and shifts in diagnostic leaf gas exchange.

## Materials and methods

### Plant culture and sampling protocols

Seedlings of *L.* *sativa* cv Paris Island and *L. serriola* were raised in growth chambers ventilated with charcoal/Purafil^®^-filtered air (CFA) as described in detail elsewhere (Balaguer et al. 1995). Six weeks after emergence, once the seventh true leaf had begun to emerge, plants were transferred to each of 3 unilluminated identical controlled environment chambers and stored in darkness for 12, 34 or 60 h. During this ‘dark treatment’ plants were exposed throughout to carbon dioxide-free air (by diverting the airflow into each chamber through a self-indicating column of soda lime) which reduced the CO_2_ concentration sufficiently to prevent stomatal closure and facilitate infiltration to enable extraction of apoplast washing fluid (AWF). After 60 h in the dark, some of the plants were transferred to growth chambers ventilated with CFA and leaf ascorbate content monitored after 2, 9, 16, and 24 h of continuous illumination at a PPFD of 400 µmol m^−2^ s^−1^. Eight plants of each species subject to each treatment were transferred to controlled environment chambers ventilated with ozone for 8 hours after 2, 9, 16 and 24 h ‘recovery’ in the light. Stomatal conductance (*g*_H2O_) was monitored during ozone exposure at 1.5 h intervals. Measurements were made in situ on the abaxial surface of the youngest fully expanded leaf of each plant using a regularly calibrated Delta-T Devices AP-4 porometer (Delta-T Devices, Cambridge, UK). Ozone concentrations inside individual controlled environment chambers were adjusted regularly to ensure equivalent ozone uptake fluxes, calculated on the basis of recorded *g*_H2O_ (see Table 1; Eq. 1).

**Table 1:** Stomatal conductance to water vapour (g_H2O_), O_3_ concentrations to which plants were exposed and calculated ozone fluxes for pre-darkened (60 h) *Lactuca sativa* and *L. serriola*, following transfer after 2, 9, and 24 h of continuous illumination (PPFD of 400 μmol m^−2^ s^−1^)

Species	2 h			9 h			24 h		
	*g*_H2O_ (mmol m^−2^ s^−1^)	O_3_ (nmol mol^−1^)	Flux (nmol m^−2^ s^−1^)	*g*_H2O_ (mmol m^−2^ s^−1^)	O_3_ (nmol mol^−1^)	Flux (nmol m^−2^ s^−1^)	*g*_H2O_ (mmol m^−2^ s^−1^)	O_3_ (nmol mol^−1^)	Flux (nmol m^−2^ s^−1^)
*L. sativa*	200 ± 33	300	36,8	144 ± 16	415	36,6	325 ± 63	185	36,9
	99 ± 10	605	36,7	79 ± 8	760	36,8	265 ± 20	225	36,5
	113 ± 16	530	36,7	82 ± 7	735	36,9	213 ± 21	280	36,6
	78 ± 11	770	36,8	73 ± 6	820	36,7	240 ± 13	250	36,8
	83 ± 16	720	36,6	85 ± 15	705	36,7	218 ± 23	275	36,7
	85 ± 12	705	36,7	92 ± 28	645	36,4	172 ± 16	350	36,9
*L. serriola*	103 ± 18	580	36,6	45 ± 6	1320	36,4	187 ± 17	320	36,7
	58 ± 7	1030	36,6	46 ± 4	1300	36,7	171 ± 36	350	36,7
	54 ± 6	1100	36,4	47 ± 4	1270	36,6	156 ± 15	385	36,8
	42 ± 11	1420	36,6	48 ± 4	1250	36,8	133 ± 9	450	36,7
	34 ± 10	1750	36,5	48 ± 5	1250	36,8	116 ± 42	515	36,6
	34 ± 6	1750	36,5	45 ± 4	1320	36,4	100 ± 10	600	36,8

Stomatal conductance was monitored during exposure to ozone at 1.5 h intervals, using a porometer. Measurements were made on the abaxial surface of the youngest fully-expanded leaf. Values for g_H2O_ represent the mean ± SE of at least six independent measurements. Atmospheric ozone concentrations inside the fumigation chambers were adjusted to ensure the delivery of equivalent ozone fluxes, calculated on the basis of recorded *g*_H2O_ (Eq. 1)

### Diagnostic leaf gas exchange analysis

The light-saturated rate of CO_2_ exchange (*A*_*sat*_; measured at a photosynthetic photon flux density—PPFD—of 1000 μmol m^−2^ s^−1^) was recorded on the most recently fully-expanded leaf (6 independent replicates per treatment) using an automated Parkinson leaf cuvette (model AUTO-PLC-B, PP Systems, Hitchin, UK) linked to an infrared gas analyzer (IRGA, model Ciras II, PP Systems, Hitchin, UK). Measurements were made at a cuvette CO_2_ concentration of 350 ± 5 μmol CO_2_ mol^−1^ dry air employing a leaf temperature of 22 ± 0.1 °C.

### Determination of ascorbate content and cell membrane integrity

Apoplast AA content and redox status was determined, with some modifications, according to Turcsànyi and co-workers (2000). Approximately 0.5 g of leaf tissue (representing one half of the most recently fully expanded leaf with the central vascular tissue removed) was vacuum-infiltrated (–80 kPa for 2–3 min for *L. sativa*; 3–4 min for *L. serriola*) with ice-cold 100 mM citric acid/potassium phosphate buffer (pH 3.0) containing 100 mM KCl and 0.2 mM DTPA. The time interval between leaf excision and the completion of infiltration was less than 10 min. Immediately after infiltration, leaves were carefully blotted dry with filter paper, reweighed, rolled and carefully inserted into conical tubes made from PTFE^®^ film. These were then placed in 1.5 ml Eppendorf tubes containing 110 μl of cold 6% (w/v) metaphosphoric acid containing 0.2 mM DTPA. Apoplast washing fluid was extracted by centrifugation (5 min, 400*g* for *L. sativa*; 5 min, 1000*g* for *L. serriola*) at 4 °C. Extracts were kept on ice, in the dark, and assayed immediately. For the determination of AA and DHA in whole leaf extracts (WLEs), *ca*. 0.1 g leaf tissue (from the mid-section of the unused half of the same leaf used for the extraction of AWF) was ground in metaphosphoric acid (6% w/v) containing 0.2 mM DTPA and extracts centrifuged at 10,000*g* for 5 min at 4 °C, prior to the recovery of the supernatant. The content of AA and DHA was determined, using the spectrophotometric method of Takahama and Oniki (1992). For AA, the absorbance of a 100 μl (50 μl for WLEs) aliquot of extract was initially measured at 265 nm in 100 mM potassium phosphate buffer (pH 6.1), then re-measured following the addition of ascorbate oxidase (AO; 5 units; EC 1.10.3.3, from *Cucurbita *sp.; Sigma). Complete oxidation of AA took no longer than 2 min. Dehydroascorbate content was determined in a separate aliquot of 100 μl (50 μl for WLEs) of extract. Initial absorbance was recorded as for AA, and then absorbance was re-measured following the addition of 40 mM DL-dithiothreitol (DTT; Sigma). Complete reduction of DHA took no longer than 10 min. Measurements were corrected to account for the absorbance of AO or DTT at 265 nm. An extinction coefficient of 14.5 mM^−1^ cm^−1^ for AA at 265 nm was used to calculate ascorbate and dehydroascorbate content (Nakano and Asada 1981).

The extent of cytoplasmic contamination of recovered AWF was assayed by measuring intracellular ‘marker’ enzyme activity (glucose-6-phosphate dehydrogenase (G6PDH) in pre-darkened, darkened and re-illuminated plants. Leaves were infiltrated with 66 mM potassium phosphate buffer (pH 7.0) containing 10 mM MgCl_2_, 100 mM KCl, 0.2 mM DTPA and 2 mg ml^−1^ DTT. Apoplast washing fluid was collected as described previously, with the modification that AWF was collected in empty Eppendorf tubes. Assays were performed immediately. For the determination of G6PDH (EC 1.1.1.49) activity in WLEs, 0.1 g leaf tissue was ground in 66 mM potassium phosphate buffer (pH 7.0) containing 10 mM MgCl_2_, 0.2 mM DTPA and 2 mg ml^−1^ DTT. Extracts were centrifuged at 10,000*g* for 3 min at 4 °C and the supernatant recovered. Supernatant was decanted into a fresh Eppendorf tube and the activity of G6PDH determined following the reduction of NADP at 340 nm at 25 °C (Kornberg and Horecker 1955). The assay contained 66 mM potassium phosphate (pH 7.6), 10 mM MgCl_2_, 6 mM NADP, 40 mM G6P (to start) and 400 μl (50 μl for WLFs). An extinction coefficient of 6.22 mM cm^−1^ for NADPH at 340 nm was used to calculate G6PDH activity.

Preliminary experimentation verified the suitability of the acidic buffer for recovery of AWF, the time of infiltration, and the centrifugal force that was optimal for AWF recovery. Optimization for the two different species was based on maximizing the return of AWF and minimizing contamination over a range of centrifugation speeds. The methods employed tend to be species-specific and maybe influenced by variety, growth conditions and leaf age (sensu Moldau et al. 1998; Lyons et al. 1999; Burkey and Eason 2002; Maddison et al. 2002; Padu et al. 2005; Burkey and Carter 2009; Feng et al. 2010). Moreover, the ease with which leaves are infiltrated and AWF recovered is also influenced by the number and size of stomata and mesophyll resistance (O'Leary et al. 2014). Measurements of G6PDH activity revealed less than 0.3% contamination of AWF by intracellular enzymes, implying the extent of cytosolic contamination of AWF was minimal.

### Gas exchange measurements/delivery of equivalent ozone flux day/night

In a separate experiment, seedlings of *L. sativa* cv Paris Island and *L. serriola* were raised as described elsewhere (Goumenaki et al. 2007). Once the fourth true leaf had begun to emerge, ten plants of each species were placed in four fully randomized blocks inside each of 12 pre-programmable controlled environment chambers. Six chambers were illuminated between 07:00 and 21:00 h. Three of them were ventilated with CFA (< 5 nmol mol^−1^ O_3_) and three received filtered air plus O_3_ for 8 h during the photoperiod (08:00–16:00). The remaining six chambers were illuminated between 17:00 and 07:00 h. Three of these were ventilated with CFA (< 5 nmol mol^−1^ O_3_) and three received filtered air plus O_3_ for 8 h during the dark period (08:00–16:00). After 5 week exposure, 10 plants of each species in each treatment were harvested and the shoots dried in an oven at 70 °C to constant weight.

Stomatal conductance (*g*_H2O_) was recorded at 4 d intervals over the fumigation period (5 weeks). Measurements were made in the middle of light or dark period, using a Delta-T porometer on the abaxial surface of the youngest fully expanded leaf of six plants (three per chamber) per variety *treatment combination, alternating between plants exposed to CFA and CFA + O_3_. To undertake measurements in the dark, a green ‘safe’ light (< 1 μmol m^−2^ s^−1^) was employed. These conditions have been shown not to affect stomatal conductance (Kim et al. 2004). Atmospheric ozone concentrations inside each fumigation chamber were adjusted to ensure the delivery of equivalent ozone fluxes, calculated on the basis of recorded *g*_H2O_ (Table 2). Ozone flux calculations took account of differences in O_3_ solubility due to lower night-time temperatures during night-time versus day-time ozone exposure (Table 2; Kosak-Channing and Heiz 1983).

**Table 2:** Stomatal conductance to water vapour (g_H2O_), temperature, O_3_ concentrations to which plants were exposed, calculated ozone solubility coefficient and ozone fluxes, during day-time *versus* night-time exposure of *Lactuca  sativa* cv Paris Island and *L. serriola*

Day-time exposure							Night-time exposure				
Species	Date	g_H2O_ (mmol m^−2^ s^−1^)	T (^o^C)	O_3_ (nmol mol^−1^)	Solubility coefficient	Flux (nmol m^−2^ s^−1^)	g_H2O_ (mmol m^−2^ s^−1^)	T (^o^C)	O_3_ (nmol mol^−1^)	Solubility coefficient	Flux (nmol m^−2^ s^−1^)
*L. sativa*	10/11	180	22	100	0.29	11.0	35	12	381	0.39	11.0
	15/11	197	22	100	0.29	12.1	85	12	172	0.39	12.1
	20/11	197	25	100	0.27	12.1	29	14	489	0.37	12.1
	25/11	179	25	100	0.27	11.0	57	13	219	0.38	11.0
	30/11	117	25	100	0.27	7.2	59	13	138	0.38	7.2
	5/12	178	24	100	0.27	10.9	100	13	128	0.38	10.9
	10/12	189	25	100	0.27	11.6	54	14	252	0.37	11.6
*L. serriola*	10/11	346	22	100	0.29	21.2	61	12	420	0.39	21.2
	15/11	160	22	100	0.29	9.8	118	12	100	0.39	9.8
	20/11	160	25	100	0.27	9.8	46	14	250	0.37	9.8
	25/11	158	25	100	0.27	9.7	40	13	276	0.38	9.7
	30/11	137	25	100	0.27	8.4	22	13	435	0.38	8.4
	5/12	111	24	100	0.27	6.8	24	13	333	0.38	6.8
	10/12	96	25	100	0.27	5.9	26	14	266	0.37	5.9

Stomatal conductance was recorded with the aid of a porometer at 4 d intervals over the fumigation period (5 weeks), on the abaxial surface of the youngest fully-expanded leaf. Values for *g*_H2O_ represent the mean of at least six independent measurements. Atmospheric ozone concentrations during night-time exposure to ozone were adjusted to ensure the delivery of equivalent day-time and night-time ozone fluxes (based on day-time exposure of 100 nmol mol^−1^ O_3_). Fluxes were calculated from recorded *g*_H2O_ (see Eq. 1)

Ozone flux was calculated using Eq. (1), whilst differences in solubility based on temperature differentials were calculated using Eq. (2):

1 $$F_{{\text{st}}} \, = \,{\text{c}}_{{\text{O3}}} *g_{{\text{H2O}}} *D_{{\text{O3}}/{\text{H2O}}} *s,$$

where *F*_st_ represents ozone uptake per unit leaf area (nmol m^−2^ s^−1^), c_O3_ is the hourly average ozone concentration (nmol m^−3^), g_H2O_ is the stomatal conductance to water vapour (m s^−1^), D_O3/H2O_ represents the difference in diffusivity between ozone and water in air (0.613; Nobel 1983), and *s* represents the ozone solubility ratio coefficient in water (pH 7):

2 $${\text{log}}_{{1}0} s\, = \,\, - \,0.{25}-0.0{13} T,$$

where *s* represents the solubility ratio coefficient in water (pH 7) and *T* is temperature ( °C).

### Statistical analyses

Statistical analyses were performed using SPSS version 20.0 (IBM, USA). Data were first subjected to ANOVA, investigating the influences of chamber on all measured parameters. No significant chamber to chamber variation was found within treatments, so data were re-analyzed under the assumption that plants in replicate chambers could be grouped within the same datasets. Two-way ANOVA was used to investigate ozone x day/night interactions. Significant differences between means were established using the least significant difference calculated at the 5% level.

## Results

### Characterization of leaf ascorbate content and redox state in plants transferred to darkness and then re-illuminated

**Fig. 1:**
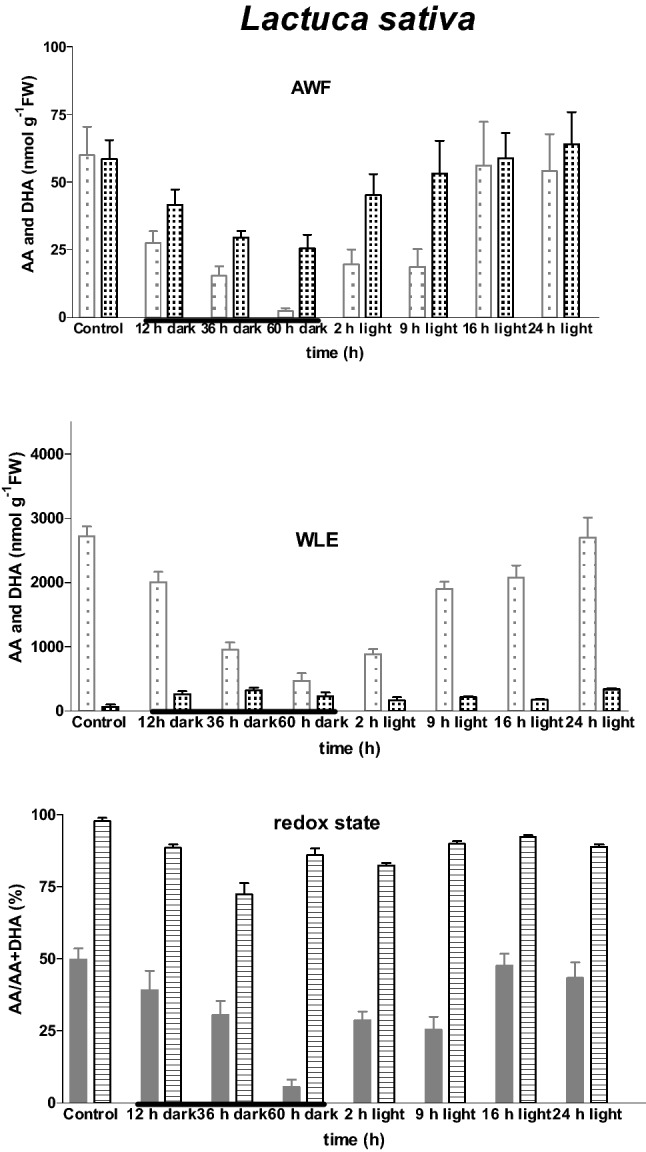
Impact of light–dark transition on leaf ascorbate content and redox status in *Lactuca sativa* cv. Paris Island. Measurements of ascorbate (AA  ) and dehydroascorbate (DHA  ) were made on apoplast washing fluid (AWF  ) and whole leaf extracts (WLEs  ) prepared using the most recent fully-expanded leaf of plants raised in controlled environment chambers. Values represent the mean (± SE) of eight independent measurements. Thickened portion of x axis indicates dark period

**Fig. 2:**
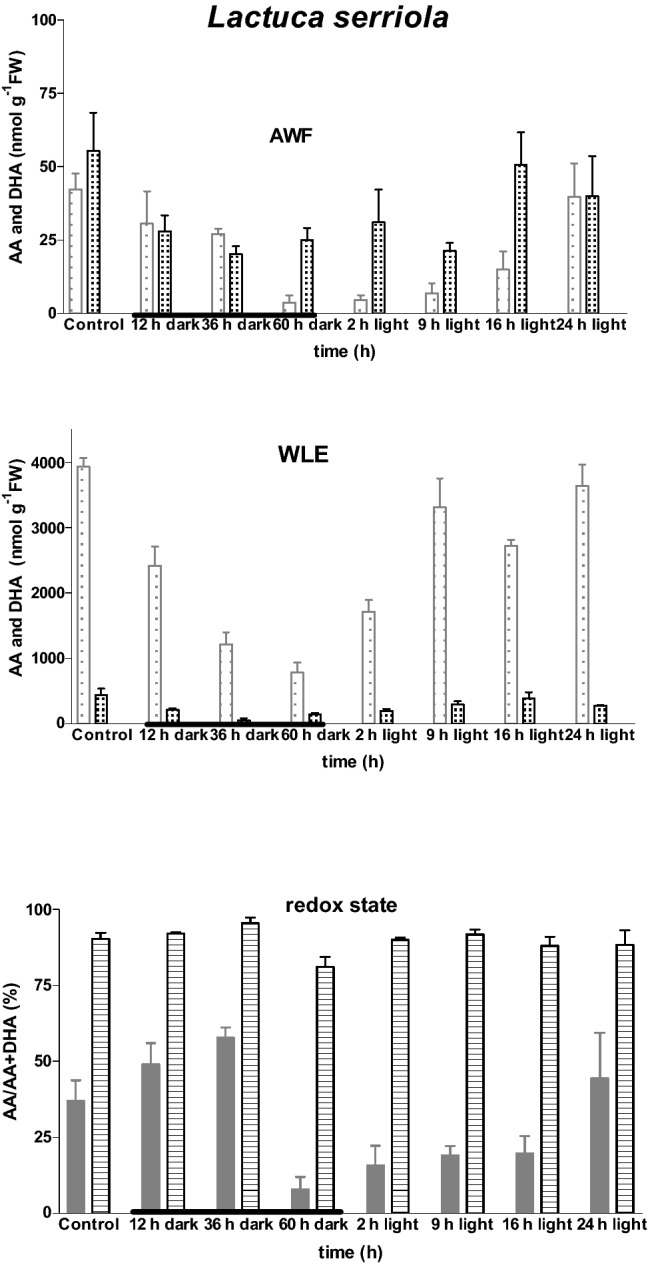
Impacts of light–dark transition on leaf ascorbate content and redox status in *Lactuca serriola.* Measurements of ascorbate (AA  ) and dehydroascorbate (DHA  ) were made on apoplast washing fluid (AWF  ) and whole leaf extracts (WLEs  ) prepared using the most recent fully-expanded leaf of plants raised in controlled environment chambers. Values represent the mean (± SE) of eight independent measurements. Thickened portion of x axis indicates dark period

Transfer of 6-week-old plants at the end of a 14-h photoperiod to darkness resulted in a marked and statistically significant (*P* < 0.001) decline in AWF and WLE AA content (in both genotypes) within 12 h of transfer. Moreover, both these AA pools continued to decline for the duration of a 60-h dark treatment. On transfer of *L. sativa* plants to the light, AA content increased rapidly and within 2 h the AA content in both AWF and WLEs had (at least) doubled in the majority of cases (Fig. 1). In contrast, AA content recovered slowly following dark–light transition in AWF in *L. serriola* (Fig. 2). Maintenance of redox state of the AA pool in the apoplast collapsed after 60 h dark-treatment in both species, but both AA content and redox state recovered to pre-dark treatment levels in both species within 24 h of transfer to the light.

The AA content of apoplast and whole leaf was closely related to the light history of the plant (Figs. 1, 2). Interestingly, the depletion of the AA pool in the apoplast appeared to be more affected by light–dark transition than the whole leaf AA pool. Analysis of variance revealed no statistically significant genotype *dark-treatment interaction, implying a similar response in both genotypes.

### Assessment of ozone impacts via determination of effects on A_sat_

**Fig. 3:**
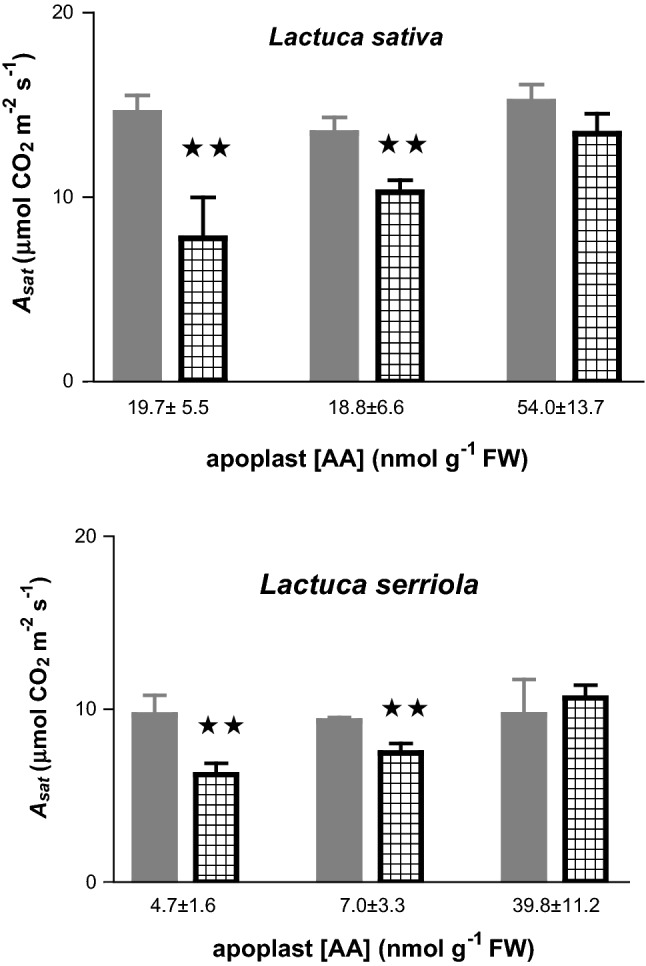
Ozone-induced decline in light-saturated rate of CO_2_ assimilation (*A*_*sat*_) in foliage of *Lactuca sativa* cv Paris Island and *Lactuca serriola* in which the apoplast pool of ascorbic acid was manipulated by the re-illumination of plants for varying periods (2, 9 or 24 h) following 60 h darkness. Plants were exposed to CFA (< 5 nmol mol^−1^ O_3_) ( ) or an O_3_ flux of 36.8 nmol m^−2^ s^−1^ (┼). Values represent the mean of six independent measurements (± SE) made on the most recently full-expanded leaf. Values on x-axis represent the mean ± SE of initial apoplast AA content. Significant differences from CFA are denoted: **P < 0.01

Figure 3 shows the extent of the decline in *A*_*sat*_ triggered by exposure to equivalent ozone flux administered 2, 9, and 24 h after re-illuminating plants that exhibit a range of AA contents and redox states. The ozone treatment (ozone flux = 36.8 nmol m^−2^ s^−1^) that was administered resulted in a significant (*P* < 0.05) decline in *A*_*sat*_ in both *L. sativa* and *L. serriola* when assayed 2 and 9 h after re-illumination. Following recovery of AWF AA levels to pre-darkness levels (after 24 h re-illumination) equivalent ozone uptake resulted in no significant effect on *A*_*sat*_.

### Assessment of ozone impacts via determination of effects on growth

**Fig. 4:**
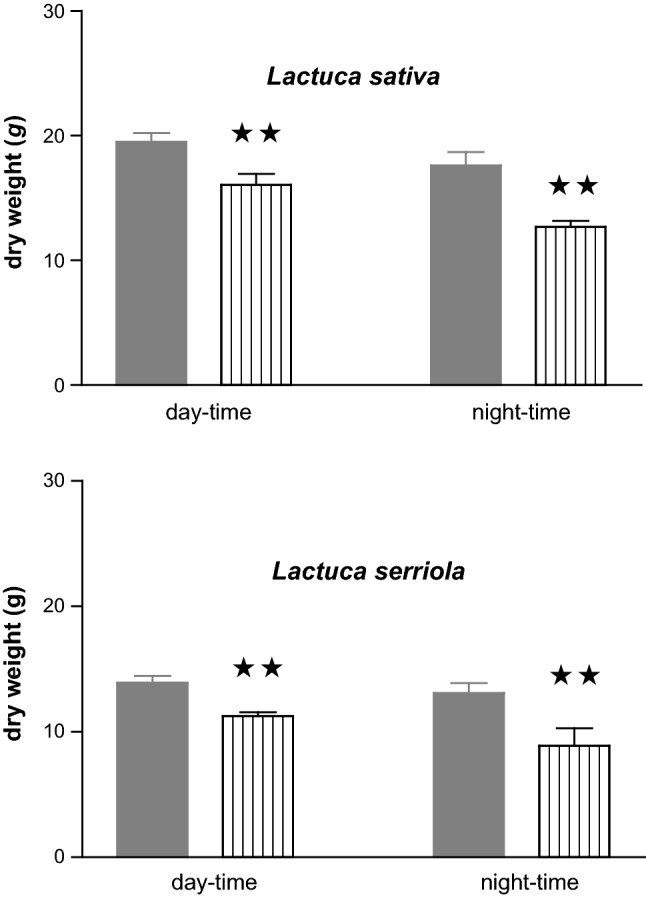
Impact of equivalent ozone uptake administered during day-time *versus* night-time on the growth of *Lactuca sativa* cv Paris Island and *L. serriola*. Plants were exposed to CFA (< 5 nmol mol^−1^ O_3_) ( ) or an O_3_ flux was calculated as shown on Table 2 ( ) based on aerial concentrations administered daily during 8 h over 5 weeks. Bars represent the mean of ten independent plants per treatment (± SE). Significant differences from CFA are denoted: **P < 0.01

*Lactuca sativa* and *L. serriola* plants exposed to equivalent ozone fluxes (Table 2) administered during the day-time *versus* night-time exhibited a significant (*P* < 0.01) decline in biomass under both conditions. Two-way ANOVA revealed a statistically significant ozone *day/night interaction (*P* < 0.01) in both species; biomass losses attributable to equivalent ozone flux were between 10% and 8% greater in *L. sativa* and *L. serriola*, respectively, when ozone was administered at night *versus* the day (Fig. 4).

## Discussion

The data reported lend further support to the contention that apoplast AA content and/or redox status govern the sensitivity of plant tissues to ozone-induced oxidative stress (Lyons et al. 1999; Plöchl et al. 2000; Robinson and Britz 2000; Barnes et al. 2002; Pignocchi et al. 2003; Conklin and Barth 2004; Feng et al. 2010; Frei et al. 2008, 2010; Fotopoulos and Kanellis 2013). Our results indicated that when plants were subjected to an ozone episode equating to a flux of 36.8 nmol m^−2^ s^−1^ the reduced ascorbate in the apoplast of *L. sativa* and *L. serriola* maybe sufficient to shield the plasmalemma and/or trigger additional defence mechanisms preventing ozone-induced oxidative damage. In contrast, under conditions, where the apoplast AA pool was depleted (i.e., following short-term re-illumination after prolonged darkness) insufficient protection was afforded so as to prevent a pronounced decline in leaf photosynthetic capacity and resulted to greater biomass losses in both species examined. The implication from these observations is that cell wall-localised AA plays a key role in defining the critical flux threshold for ozone damage.

By carefully managing the ozone exposure in conjunction with measurements of stomatal conductance, it was possible to administer equivalent ozone fluxes during the day and night-time. Ozone concentrations employed at night were unnaturally high, not related to existing or foreseen atmospheric levels, but these concentrations were necessary to achieve equivalent fluxes day and night, given the differences in stomatal conductance under the different conditions, to test the hypothesis under investigation.

Our experimental approach revealed that plants are affected significantly more by equivalent ozone flux taken-up at night compared to during the day. This finding is consistent with the theory that detoxification potential is compromised at night (Matyssek et al. 1995; Wieser and Havranek 1995; Musselman and Minnick 2000; Lloyd et al. 2018; Foyer et al. 2020). Our conclusions, that night-time depletion in cell wall-localised ascorbate content and/or redox status may enhance the ‘sensitivity’ of plants to ozone at night time, are consistent with findings reported by other authors (Moldau et al. 1998; Plöchl et al. 2000; Turcsànyi et al. 2000; Maddison et al. 2002; Burkey and Eason 2002; Sanmartin et al. 2003; Padu et al. 2005; Höller et al 2015). It should not, however, be overlooked that the prolonged dark treatment employed to manipulate AA levels in the present study, as in many others, may trigger a suite of alterations in metabolism in addition to the targeted shift in AA levels (Poór et al. 2018). Such pleiotropic effects were not examined in the present study but warrant further investigation.

In this study *L. sativa* showed higher biomass loss and higher nocturnal conductance, when exposed to equivalent ozone fluxes at day-time versus night-time, compared to the closest wild species *L. serriola*. The two species have been shown to exhibit different morphological and physiochemical characteristics, that are reflected in the phyllosphere bacterial community, which can influence plant responses to biotic stress (Hunter et al. 2010; Bailey et al. 2019). Leaf characteristics have been correlated with differential ozone responses in other species (Calatayud et al. 2011; Li et al. 2016). Factors governing the differential response to O_3_ of the two *Lactuca* species used in this study remain to be established (see Goumenaki and Barnes, 2009; Goumenaki et al. 2010).

Incomplete stomatal closure at night has been observed in a diverse range of C_3_ and C_4_ species (Caird et al. 2007), and night-time exposure can cause significant damage to sensitive plants under some conditions (Musselman and Minnick 2000; Grulke et al. 2004; Mereu et al. 2009; González-Fernández et al. 2010). Extensive endeavours to map the risks posed by ozone pollution to terrestrial vegetation are understandably focussed on cumulative stomatal flux during day-time. The results of the present study suggest that some thought should be given to weighing regional models of ozone impacts for extraordinary night-time ozone impacts, since these results showed that from a flux-perspective plants are more sensitive to ozone taken up into foliage at night. The notion that tropospheric ozone metrics used for risk assessment to vegetation should include night-time exposures is also supported by other studies (Musselman and Minnick 2000; Grulke et al. 2004; Mereu et al. 2009; Mills et al. 2018; Hoshika et al. 2019) and could be especially important for the protection of species and communities which often show significant conductance at night, e.g. wetland plant communities and many common tree species. Our findings are consistent with a role for diel shifts in apoplast AA content and/or redox status determining the reaction of plant tissues to ozone-induced oxidative stress. Depletion of the AA pool in the apoplast alters ‘sensitivity’ to ozone in a manner that is predictably based on diffusional limitations and biochemical reactions following the uptake of the gas into the leaf interior. Future work is needed to explore the relationship between stress and antioxidants on a quantitative basis in more detail and incorporate detoxification capacity in improved flux-effect models employed for ozone risk assessment.

## Conclusions

The data presented show that apoplast AA content and/or redox state is subject to day/night control. The adopted experimental approach enabled manipulation of leaf apoplast and symplast ascorbate content to test whether such experimental manipulations are linked with shifts in tolerance to short term (acute) O_3_ exposure. Plants exposed to equivalent ozone fluxes administered during day-time versus night-time exhibited a significant decline in biomass in both cases, and these losses were greater in plants subjected to equivalent ozone flux at night. This observation was consistent with the night-time depletion of cell wall-localised ascorbate.

### *Author contribution statement*

EG and JB conceived and designed the research. EG conducted the experiments. EG, IGF and JB analyzed data. EG wrote the manuscript with contributions from all the authors.
